# CVOT Summit Report 2023: new cardiovascular, kidney, and metabolic outcomes

**DOI:** 10.1186/s12933-024-02180-8

**Published:** 2024-03-19

**Authors:** Oliver Schnell, Katharine Barnard-Kelly, Tadej Battelino, Antonio Ceriello, Helena Elding Larsson, Beatriz Fernández-Fernández, Thomas Forst, Juan-Pablo Frias, James R. Gavin, Francesco Giorgino, Per-Henrik Groop, Hiddo J. L. Heerspink, Stephan Herzig, Michael Hummel, George Huntley, Mahmoud Ibrahim, Baruch Itzhak, Stephan Jacob, Linong Ji, Mikhail Kosiborod, Nebosja Lalic, Sofia Macieira, Rayaz A. Malik, Boris Mankovsky, Nikolaus Marx, Chantal Mathieu, Timo D. Müller, Kausik Ray, Helena W. Rodbard, Peter Rossing, Lars Rydén, Petra-Maria Schumm-Draeger, Peter Schwarz, Jan Škrha, Frank Snoek, Frank Tacke, Bruce Taylor, Britta Tendal Jeppesen, Solomon Tesfaye, Pinar Topsever, Tina Vilsbøll, Xuefeng Yu, Eberhard Standl

**Affiliations:** 1 Forschergruppe Diabetes e. V, Helmholtz Center Munich, Ingolstaedter Landstraße 1, 85764 Neuherberg (Munich), Germany; 2 Southern Health NHS Foundation Trust, Southampton, United Kingdom; 3 University Medical Center, Ljubljana, Slovenia; 4 Faculty of Medicine, University of Ljubljana, Ljubljana, Slovenia; 5 IRCCS MultiMedica, Milan, Italy; 6 Department of Pediatrics, Skåne University Hospital, Malmö/Lund, Sweden; 7 Department of Clinical Sciences Malmö, Lund University, Lund, Sweden; 8 Division of Nephrology and Hypertension, University Hospital Fundación Jiménez Díaz, Madrid, Spain; 9 CRS Clinical Research Services Mannheim GmbH, Mannheim, Germany; 10 Biomea Fusion, Redwood City, CA United States of America; 11 Emory University School of Medicine, Atlanta, GA United States of America; 12 Department of Precision and Regenerative Medicine and Ionian Area, University of Bari Aldo Moro, Bari, Italy; 13 Department of Nephrology, University of Helsinki and Helsinki University Hospital, Helsinki, Finland; 14 Department of Diabetes, Central Medical School, Monash University, Melbourne, Australia; 15 Department of Clinical Pharmacy and Pharmacology, University Medical Center Groningen, University of Groningen, Groningen, The Netherlands; 16 Division Diabetic Complications, Institute for Diabetes and Cancer, Helmholtz Center Munich, Neuherberg, Germany; 17 Diabetes Leadership Council, Indianapolis, IN United States of America; 18 Center for Diabetes Education, EDC, Charlotte, NC United States of America; 19 Clalit Health Services, Haifa, Israel; 20 Technion Faculty of Medicine, Haifa, Israel; 21 Practice for Prevention and Therapy and Cardio-Metabolic Institute, Villingen-Schwenningen, Germany; 22 Peking University People’s Hospital, Xicheng District, Beijing, China; 23 Department of Cardiovascular Disease, Saint Luke’s Mid America Heart Institute, University of Missouri-Kansas City School of Medicine, Kansas City, MO United States of America; 24 Clinic for Endocrinology, Diabetes and Metabolic Diseases, University Clinical Center of Serbia, Belgrade, Serbia; 25 Faculty of Medicine, University of Belgrade, Belgrade, Serbia; 26 Sciarc GmbH, Baierbrunn, Germany; 27 Weill Cornell Medicine-Qatar, Qatar Foundation-Education City, Ar-Rayyan, Doha, Qatar; 28 Shupyk National Healthcare University of Ukraine, Kyiv, Ukraine; 29 Clinic for Cardiology, Pneumology, Angiology and Internal Intensive Care Medicine (Medical Clinic I), RWTH Aachen University Hospital, Aachen, Germany; 30 Department of Endocrinology, Catholic University Leuven, Leuven, Belgium; 31 Institute for Diabetes and Obesity, Helmholtz Munich, Munich, Germany; 32 Walther-Straub Institute for Pharmacology and Toxicology, Ludwig-Maximilians-University Munich (LMU), Munich, Germany; 33 School of Public Health, Imperial College London, London, United Kingdom; 34 Endocrine and Metabolic Consultants, Rockville, MD United States of America; 35 Steno Diabetes Center Copenhagen, Herlev, Denmark; 36 Department of Clinical Medicine, University of Copenhagen, Copenhagen, Denmark; 37 Department of Medicine K2, Karolinska Institute, Stockholm, Sweden; 38 Center Internal Medicine Fünf Höfe, Munich, Germany; 39 Medical Clinic III, University Hospital Carl Gustav Carus, Dresden, Germany; 40 Third Medical Department and Laboratory for Endocrinology and Metabolism, First Faculty of Medicine, Charles University, Prague, Czech Republic; 41 Amsterdam University Medical Centers, Amsterdam, The Netherlands; 42 Department of Hepatology and Gastroenterology, Charité Universitätsmedizin Berlin, Campus Virchow-Klinikum and Campus Charité Mitte, Berlin, Germany; 43 Diabetes Patient Advocacy Coalition, Tampa, FL United States of America; 44 Future Evidence Foundation, Melbourne, Australia; 45 Sheffield Teaching Hospitals, Sheffield, United Kingdom; 46 University of Sheffield, Sheffield, United Kingdom; 47 Department of Family Medicine, Acıbadem Mehmet Ali Aydınlar University School of Medicine, Istanbul, Türkiye; 48 Division of Endocrinology, Department of Internal Medicine, Tongji Hospital, Tongji Medical College, Huazhong University of Science and Technology, Wuhan, China

**Keywords:** Cardiovascular disease, Chronic kidney disease, CGM, Diabetes, Finerenone, GLP-1 RA, Guidelines, Heart failure, MASLD, NAFLD, Obesity, SGLT2 inhibitor, Teplizumab

## Abstract

The 9th Cardiovascular Outcome Trial (CVOT) Summit: Congress on Cardiovascular, Kidney, and Metabolic Outcomes was held virtually on November 30-December 1, 2023. This reference congress served as a platform for in-depth discussions and exchange on recently completed outcomes trials including dapagliflozin (DAPA-MI), semaglutide (SELECT and STEP-HFpEF) and bempedoic acid (CLEAR Outcomes), and the advances they represent in reducing the risk of major adverse cardiovascular events (MACE), improving metabolic outcomes, and treating obesity-related heart failure with preserved ejection fraction (HFpEF). A broad audience of endocrinologists, diabetologists, cardiologists, nephrologists and primary care physicians participated in online discussions on guideline updates for the management of cardiovascular disease (CVD) in diabetes, heart failure (HF) and chronic kidney disease (CKD); advances in the management of type 1 diabetes (T1D) and its comorbidities; advances in the management of CKD with SGLT2 inhibitors and non-steroidal mineralocorticoid receptor antagonists (nsMRAs); and advances in the treatment of obesity with GLP-1 and dual GIP/GLP-1 receptor agonists. The association of diabetes and obesity with nonalcoholic steatohepatitis (NASH; metabolic dysfunction-associated steatohepatitis, MASH) and cancer and possible treatments for these complications were also explored. It is generally assumed that treatment of chronic diseases is equally effective for all patients. However, as discussed at the Summit, this assumption may not be true. Therefore, it is important to enroll patients from diverse racial and ethnic groups in clinical trials and to analyze patient-reported outcomes to assess treatment efficacy, and to develop innovative approaches to tailor medications to those who benefit most with minimal side effects. Other keys to a successful management of diabetes and comorbidities, including dementia, entail the use of continuous glucose monitoring (CGM) technology and the implementation of appropriate patient-physician communication strategies. The 10th Cardiovascular Outcome Trial Summit will be held virtually on December 5–6, 2024 (http://www.cvot.org).

## Background

The CVOT Summit is an annual gathering and discussion forum held in Munich, Germany, and broadcast online. Its primary focus is on the data released annually from cardiovascular, kidney, and metabolic outcomes trials, and it showcases the latest treatment innovations in the areas of diabetes, obesity, CVD, CKD, nonalcoholic fatty liver disease (NAFLD; metabolic dysfunction associated steatotic liver disease, MASLD) and NASH (MASH).

Diabetes and obesity are metabolic diseases of increasing global prevalence and concern. The International Diabetes Federation (IDF) estimates that the number of people with diabetes will increase from 537 million (10.5%) in 2021 to 783.2 million (12.2%) in 2045 [1] and according to the estimates of the World Obesity Federation, more than the half of the world population will have overweight or obesity by the year 2035 [2]. Both conditions are independently associated with an increased risk of cardiovascular (CV) complications and diseases, which can be the cause of death for at least half of individuals with type 2 diabetes (T2D) [3] and more than two thirds of individuals with high body mass index (BMI) [4].

Uncertainty about the CV safety of the thiazolidinedione rosiglitazone [5], a glucose-lowering medication approved for the treatment of T2D by the U.S. Food and Drug Administration (FDA), sparked controversy and prompted the FDA to update its guidance to industry in 2008 [6], requiring that all new T2D therapies be evaluated in long-term CVOTs. Since then, new classes of glucose-lowering drugs have been introduced for the treatment of T2D, namely di-peptidyl peptidase 4 inhibitors (DPP-4is), sodium-glucose cotransporter-2 inhibitors (SGLT2is), glucagon-like peptide 1 receptor agonists (GLP-1 RAs) and, since 2022, tirzepatide, a dual glucose-dependent insulinotropic polypeptide (GIP)/ GLP-1 RA. Finerenone, a nsMRA, has also been developed and approved for patients with T2D and CKD.

CKD and HF are major complications of diabetes. CKD develops in at least 40% of people with T2D [7, 8], reducing their life expectancy by 16 years [9], whereas HF affects 10–30% of all subjects with T2D [10]. Therefore, not only CVOTs, but also kidney outcome and HF trials have been conducted with these new medications. Until 2022, five and six CVOTs have been conducted for DPP-4is [11–15] and GLP-1 RAs [16–21], respectively, and five CVOTs [22–26], five HF [27–31] and three kidney [32–34] outcome trials with SGLT2is were published. The RENAL LIFECYCLE trial (NCT05374291) is currently evaluating the CV and kidney outcomes with dapagliflozin (SGLT2i) in patients with severe CKD. Regarding finerenone, one CVOT [35] and one kidney outcome trial [36] have been completed and one HF study, FINEARTS-HF (NCT04435626), is ongoing. In addition, FIND-CKD (NCT05047263) is evaluating its effects in non-diabetic CKD patients, and CAPTIVATE-CKD (NCT06058585) is evaluating the best finerenone treatment or combination of treatments to slow CKD progression. In the case of tirzepatide, two CVOTs are currently being conducted: SURPASS-CVOT (NCT04255433) in people with T2D and a history of CVD and SURMOUNT-MMO (NCT05556512) in people who are overweight or living with obesity. Tirzepatide is also being evaluated in HF and CKD in the SUMMIT (NCT04847557) and TREASURE-CKD (NCT05536804) trials, respectively.

Typically, CVOTs with DDP-4is, GLP-1RAs, SGLT2is and finerenone included a three-point composite of major adverse cardiovascular events (3P-MACE): CV death, nonfatal myocardial infarction (MI), and nonfatal stroke. DPP-4is were noninferior to placebo in the 3P-MACE [11–15]. In addition, CV benefits, including reduction in the number of hospitalizations for heart failure (HHF), have been observed with SGLT2is [22–31], GLP-1 RAs [16–21], and finerenone [35–37]. A further CVOT, DAPA-MI [38] investigating the effect of dapagliflozin (SGLT2i) in patients hospitalized for MI, but without prior diabetes or chronic symptomatic HF was completed in 2023.

Beyond the glucose-lowering benefits, significant effects on weight reduction were shown in trials with semaglutide (GLP-1RA) [39–42] and tirzepatide (GIP/GLP-1 RA) [43–48] in people with or without T2D. In 2023, this list of trials was augmented by one CVOT, the SELECT trial [49], and one HF trial, STEP-HFpEF [50], with semaglutide in people with overweight or obesity but without diabetes, as based on HbA1c determinations, though some 50% of participants were in the dysglycemic range.

Both diabetes and obesity are often accompanied by atherogenic dyslipidemia, characterized by elevated triglycerides, low high-density lipoprotein cholesterol (HDL-C) level and normal-to-mildly elevated low-density lipoprotein cholesterol (LDL-C) [51]. The first-line therapy for lowering LDL-C remains statins, which reduce cholesterol synthesis by inhibiting 3-hydroxy-3-methylglutaryl coenzyme A reductase (HMC-CoA reductase) and have demonstrated clear efficacy in preventing CV events and reducing CV mortality [52]. However, 7–29% of patients complain about statin-associated muscle symptoms [53].

In contrast to statins, bempedoic acid is a prodrug activated in the liver and not in the peripheral tissues. This compound reduces cholesterol synthesis by inhibiting ATP citrate lyase, upstream of HMG-CoA reductase [54]. In 2023, the results of the CLEAR Outcomes trial, a CVOT evaluating the effects of bempedoic acid on adverse CV events in people at high risk for CVD, were published [55].

Following the practice of previous years [56–63], we present and summarize the key aspects discussed at the 9th CVOT Summit: Congress on Cardiovascular, Kidney, and Metabolic Outcomes held virtually on November 30- December 1, 2023. The Summit was an interdisciplinary platform organized in collaboration with five study groups: Primary Care Diabetes Europe (PCDE, www.pcdeurope.org), Diabetes and Cardiovascular Disease EASD Study Group (DCVD, www.dcvd.org), European Diabetic Nephropathy Study Group (EDNSG, www.ednsg.org) the European Incretin Study Group (www.increti-studygroup.ch) and the Working Group Diabetes & Herz (www.ddg.org). An international audience of specialists in diabetology, endocrinology, cardiology, nephrology, and primary care from 45 countries joined the speakers and contributed to the discussions at the CVOT Summit on Cardiovascular, Kidney and Metabolic Outcomes 2023 (www.cvot.org).

## Updates on CVOTs

A summary of the characteristics and results of CV and HF outcome trials published in 2023 is listed in Tables 1, 2, 3 and 4.

## SGLT2 inhibitors

### DAPA-MI

The DAPA-MI trial [38] investigated the effect of dapagliflozin (10 mg/daily) when added to standard of care in 4017 patients hospitalized for MI, with impaired left ventricular (LV) systolic function or Q-wave MI, but without prior diabetes or chronic symptomatic HF. Participants were eligible if they were ≥ 18 years old, clinically stable, and hospitalized for acute MI, including ST-segment elevation MI (STEMI) and non-STEMI [38]. The exclusion criteria included an established diagnosis of diabetes, chronic symptomatic HF with HHF within the last year associated with a LVEF ≤ 40% and current treatment with an SGLT2 inhibitor. Patients were assigned to receive either dapagliflozin (*n* = 2019) or placebo (*n* = 1998).

The primary outcome was defined as a hierarchical composite of seven components: death, HHF, nonfatal MI, atrial fibrillation/flutter, T2D, New York Heart Association (NYHA) functional classification at the last visit, and body weight decrease ≥ 5% at the last visit. The key secondary outcome was the same composite as the primary outcome excluding body weight. Other secondary endpoints included time to the first occurrence of CV death or HHF [38].

The treatment and the placebo group did not differ in the baseline characteristics. On admission, 9.1% of the patients had prior MI, 2.4% had prior stroke and 72% had STEMI. The mean HbA1c at index hospitalization was 5.7% (39 mmol/mol) [38].

The differences in the primary and the key secondary outcomes of the dapagliflozin and placebo group were analyzed with the win ratio method [64]. The other secondary endpoints were analyzed with Cox-proportional-hazards models.

The DAPA-MI estimated trial duration was 30 months, and the statistical analysis included data from patients with at least three months of follow-up. The primary hierarchical seven-component composite outcome resulted in 32.9% wins for dapagliflozin and 24.6% wins for placebo (win ratio (WR) 1.34; 95% CI 1.20 to 1.50; *p* < 0.001) (Table 1) [38]. The key secondary outcome including six of the components resulted in 20.3% wins for dapagliflozin and 16.9% wins for placebo (WR 1.20; 95% CI 1.04 to 1.40; *p* = 0.015) (Table 1) [38]. Overall, treatment with dapagliflozin compared to placebo resulted in a significant benefit in cardiometabolic outcomes (34% more wins in the primary outcome and 20% more wins in the key secondary outcome).

The rates of the composite time to CV death or HHF (other secondary endpoints) were similar in the two treatment groups: 2.5% (*n* = 50) in the dapagliflozin group vs. 2.6% (*n* = 52) in the placebo group (HR 0.95; 95% CI 0.64 to 1.40) (Table 1) [38] and were not considered statistically significant. The rates of other prespecified secondary endpoints listed in Table 1 were low, with differences between groups not reaching nominal statistical significance.

No safety concerns were reported. Serious adverse advents on treatment leading to death occurred in 1.5% (*n* = 30) in the dapagliflozin group and in 1.5% (*n* = 29) in the placebo group [38].

**Table 1:** Key information of the DAPA-MI trial [38]

DAPA-MI		
**Class &** **Cardiovascular (CV) outcomes**	**WR (95% CI)**	***p-value***‡
**Primary outcome**		
Composite of death, HHF, nonfatal MI, AF/flutter, T2D, NYHA class, body weight decrease ≥ 5%	1.34 (1.20 to 1.50)	< 0.001
**Key secondary outcome**		
Composite of death, HHF, nonfatal MI, AF/flutter, T2D, NYHA class	1.20 (1.04 to 1.40)	0.015
**Other secondary endpoints**	**HR (95% CI)**	
Composite of CV death/HHF	0.95 (0.64 to 1.40)	
CV death/HHF/MI	0.95 (0.70 to 1.29)	
MACE (MI, stroke, or CV death)	0.94 (0.67 to 1.31)	
All-cause death	1.22 (0.77 to 1.92)	
CV death	1.15 (0.66 to 2.01)	
MI	1.11 (0.72 to 1.71)	
Stroke	0.61 (0.28 to 1.34)	
New diagnosis of T2D	0.53 (0.36 to 0.77)	
New diagnosis of AF/flutter	0.88 (0.45 to 1.73)	
All-cause hospitalization	1.12 (0.97 to 1.29)	
Adjudicated HHF	0.83 (0.50 to 1.39)	

‡ Only significant p-values are presented

AF, atrial fibrillation; CI, confidence interval; CV, cardiovascular; HF, heart failure; HHF, hospitalization for HF; HR, hazard ratio; MACE, major adverse cardiovascular event; MI, myocardial infarction; NYHA, New York Heart Association; T2D, type 2 diabetes; WR, win ratio

## GLP-1 receptor agonists

### SELECT

The SELECT trial [49] studied the effect of subcutaneous semaglutide (2.4 mg/weekly) on adverse CV events in patients with overweight or obesity without diabetes. A total of 17,604 patients, ≥ 45 years old, with a BMI ≥ 27 and established CVD were enrolled and randomly assigned to semaglutide (*n* = 8803) or placebo (*n* = 8801). CVD was defined as previous MI, previous stroke, or symptomatic peripheral arterial disease, but participants were ineligible if they were within 60 days of a CV or neurological event or if they were scheduled to undergo coronary, carotid, or peripheral revascularization. Other key exclusion criteria included a previous diagnosis of diabetes, HbA1c ≥ 6.5% (48 mmol/mol), treatment with GLP-1 RA or any glucose-lowering medication within the previous 90 days, NYHA functional class IV, or end stage kidney disease or dialysis [49].

Over 75% of the patients had had a previous MI and approximately 25% had chronic HF. Most of the patients were receiving lipid-lowering medications (90.1%), platelet-aggregation inhibitors (86.2%) and beta blockers (70.2%) [49]. Additionally, 45% of the patients were taking angiotensin-converting-enzyme inhibitors (ACEis) and 29.5% were taking angiotensin-receptor blockers (ARBs) [49].

The primary efficacy endpoint, assessed in a time-to first-event analysis, was a 3P-MACE: death from CV causes, nonfatal MI, or nonfatal stroke [49]. The confirmatory secondary endpoints, assessed in a time-to first-event analysis and tested in hierarchical order, were death from CV causes, a composite HF endpoint with three components, including death from CV causes or HHF or an urgent medical visit for HF, and death from any cause [49]. Additional secondary endpoints have been defined and described [49].

The mean duration of exposure to semaglutide and placebo in the overall trail population was 33.3 and 35.1 months, respectively. The target dose of 2.4 mg of semaglutide was achieved gradually after 16 weeks of treatment, with the starting dose being 0.24 mg once weekly, increased every 4 weeks to once weekly doses of 0.5, 1.0, 1.7 and 2.4 mg [49].

The endpoints were analyzed using a Cox proportional hazards regression model. Treatment with semaglutide for approximately 33 months resulted in a mean body weight loss of 9.4% and a 20% reduction of the 3P-MACE composite risk (HR 0.80; 95% CI 0.72 to 0.90; *p* < 0.001) (Table 2) [49]. Death from CV causes, the first confirmatory second endpoint, occurred in 2.5% (*n* = 223) of the patients in the semaglutide group and in 3.0% (*n* = 262) of the patients of the placebo group (HR 0.85; 95% CI 0.71 to 1.01; *p* = 0.07) (Table 2) and did not meet the required p-value for hierarchical testing [49]. Therefore, between-group differences were not reported for the subsequent secondary endpoints, including the composite of HF (HR 0.82; 95% CI 0.71 to 0.96) and death from any cause (HR 0.81; 95% CI 0.71 to 0.93) (Table 2).

The incidence of serious adverse advents was lower in the semaglutide group than in the placebo group (Table 2). Serious adverse events, including cardiac disorders, infections and infestations, nervous system disorders, surgical and medical procedures, neoplasms benign, malignant and unspecified, and gastrointestinal disorders were reported in 33.4% (*n* = 2941) patients of the semaglutide group and 36.4% (*n* = 3204) patients of the placebo group (*p* < 0.001) (Table 2) [49]. However, the number of adverse events leading to treatment discontinuation was higher in the semaglutide group (16.6%; *n* = 1461) than in the placebo group (8.2%; *n* = 718) (*p* < 0.001) (Table 2). These events included gastrointestinal disorders such as nausea, vomiting and diarrhea reported by 10.0% (*n* = 880) of the patients in the semaglutide group vs. 2.0% (*n* = 172) in the placebo group (*p* < 0.001). Gallbladder-related disorders were reported by 2.8% (*n* = 246) patients in the semaglutide group and 2.3% (*n* = 203) patients in the placebo group (*p* = 0.04) (Table 2) [49]. The incidence of other adverse events of interest is shown in Table 2.

**Table 2:** Key information of the SELECT trial [49]

SELECT		
**Class &** **Cardiovascular (CV) outcomes**	**HR (95% CI)**	***p-value***‡
**Primary efficacy endpoint**		
Composite of 3P-MACE: CV death, nonfatal MI, or nonfatal stroke	0.80 (0.72 to 0.90)	< 0.001
**Confirmatory secondary efficacy endpoints**		
CV death	0.85 (0.71 to 1.01)	0.07
Composite of HF: CV death, HHF, or urgent medical visit for HF	0.82 (0.71 to 0.96)	
Death from any cause	0.81 (0.71 to 0.93)	
**Adverse events**	**Event rate (%) active vs. placebo group**	***p-value***
**Serious adverse events**	33.4 vs. 36.4	< 0.001
Cardiac disorders	11.5 vs. 13.5	< 0.001
Infections and infestations	7.1 vs. 8.4	0.001
Nervous system disorders	5.0 vs. 5.6	0.08
Surgical and medical procedures	4.9 vs. 6.2	< 0.001
Neoplasms benign, malignant, and unspecified	4.6 vs. 4.6	0.94
Gastrointestinal disorders	3.9 vs. 3.7	0.48
**Adverse events leading to treatment discontinuation**	16.6 vs. 8.2	< 0.001
Gastrointestinal disorders	10.0 vs. 2.0	< 0.001
Nervous system disorders	1.4 vs. 1.0	0.03
Metabolism and nutrition disorders	1.2 vs. 0.3	< 0.001
General disorders and administration-site conditions	1.2 vs. 0.5	< 0.001
Neoplasms benign, malignant, and unspecified	0.9 vs. 1.2	0.07
Infections and infestations	0.9 vs. 1.0	0.47
**Other adverse events of interest**		
Gallbladder-related disorders	2.8 vs. 2.3	0.04
Acute kidney failure	1.9 vs. 2.3	0.13
Acute pancreatitis	0.2 vs. 0.3	0.28

‡ p-values after the first nonsignificant p-value are not presented

CI, confidence interval; CV, cardiovascular; HF, heart failure; HHF, hospitalization for HF; HR, hazard ratio; MACE, major adverse cardiovascular event; MI, myocardial infarction

### STEP-HFpEF trial

The STEP-HFpEF trial [50] evaluated the safety and efficacy of semaglutide (2.4 mg/weekly) injected subcutaneously in 529 patients with heart failure with preserved ejection fraction (HFpEF) and obesity, without diabetes. Participants ≥ 18 years old were eligible if they had a LVEF ≥ 45%, a BMI ≥ 30, NYHA functional class II, III, or IV symptoms, a Kansas City Cardiomyopathy Questionnaire clinical summary score (KCCQ-CSS) < 90 points, a 6-minute walk distance of at least 100 m, and a least one of the following: elevated left ventricular filling pressures (invasively measured) or elevated natriuretic peptides (stratified according to BMI at baseline) plus echocardiographic abnormalities, or HHF in the previous 12 months plus ongoing treatment with diuretics or echocardiographic abnormalities [50]. Key exclusion criteria were a patient self-reported weight change > 5 Kg within 90 days before screening, HbA1c ≥ 6.5% (48 mmol/mol) or a known history of diabetes.

Participants were randomly assigned to receive semaglutide (*n* = 263) or placebo (*n* = 266) for 52 weeks followed by a 5-week follow-up period. The target dose of 2.4 mg of semaglutide was achieved gradually after 16 weeks of treatment, with the starting dose of semaglutide being 0.25 mg once weekly, increased every 4 weeks to once weekly doses of 0.5, 1.0, 1.7 and 2.4 mg [50].

Dual primary endpoints were established: a change in the KCCQ-CSS and the percentage change in body weight from baseline to week 52. Three confirmatory secondary endpoints were defined: the 6-minute walk distance from baseline to week 52, a hierarchical composite endpoint and a change in the level of C-reactive protein (CRP) from screening (week − 2) to week 52. The hierarchical composite endpoint included death from any cause from baseline to week 57, the number and timing of HF events requiring hospitalization or urgent HF visit from baseline to week 57, differences of at least 15, 10, or 5 points in KCCQ-CSS change between baseline and week 52 and a difference of at least 30 m in the change in the 6-minute walk distance from baseline to week 52 [50]. Supportive secondary endpoints have also been described [50].

The dual primary and confirmatory secondary endpoints were assessed for all randomized patients using the intend-to-treat principle (treatment policy estimand). Continuous endpoints in this trial were evaluated using analysis of covariance [50]. The hierarchical composite endpoint was evaluated using the win ratio approach [64]. The CRP levels in the two groups were compared by first estimating the geometric mean ratio of the week 52 value to the baseline value for each group, and then calculating the ratio between the values obtained for the semaglutide and placebo groups [50].

The mean change in KCCQ-CSS at week 52, one of the primary endpoints, was 16.6 points in the semaglutide group and 8.7 points in the placebo group (estimated difference 7.8 points; 95% CI 4.8 to 10.9; *p* < 0.001) (Table 3). The other dual primary endpoint, the mean percentage change in body weight at week 52 was − 13.3% in the semaglutide group and − 2.6% in the placebo group (estimated difference − 10.7%; 95% CI -11.9 to -9.4; *p* < 0.001) (Table 3).

Analysis of the confirmatory secondary endpoints resulted in a mean change in the 6-minute walk distance at week 52 of 21.5 m in the semaglutide group and 1.2 m in the placebo group (estimated difference 20.3 m; 95% CI 8.6 to 32.1; *p* < 0.001) (Table 3). Participants in the semaglutide group had a 43.5% reduction in CRP level at 52 weeks (geometric mean ratio [week 52 value to baseline value], 0.56), as compared with a 7.3% reduction with placebo (geometric mean ratio [week 52 value to baseline value], 0.93) (estimated treatment ratio 0.61; 95% CI, 0.51 to 0.72; *p* < 0.001) [50]. The results of the hierarchical composite secondary endpoint comparing all participants in the semaglutide and placebo groups within each BMI stratum (< 35 and ≥ 35) revealed a score of 60.1 wins for the semaglutide group and 34.9 wins for the placebo group (stratified WR 1.72; 95% CI 1.37 to 2.15; *p* < 0.001), with a difference of at least 15 points in the change in KCCQ-CSS contributing the most wins for semaglutide (Table 3) [50].

Serious adverse events were reported in 13.3% (*n* = 35) of the participants in the semaglutide group and 26.7% (*n* = 71) participants in the placebo group (*p* < 0.001)(Table 3) [50] and were mainly attributed to cardiac disorders, which were reported in 2.7% (*n* = 7) of the patients in the semaglutide group and in 11.3% (*n* = 30) of the patients in the placebo group (*p* < 0.001) (Table 3). Gastrointestinal disorders were similarly reported with an incidence of 2.7% (*n* = 7) in the semaglutide group and of 2.6% (*n* = 79 in the placebo group (*p* = 1.00) (Table 3). These events were the most common reason for discontinuation in both the semaglutide (*n* = 6) and placebo (*n* = 6) groups.

Death from any cause, death from CV causes and HF event were adjudicated as adverse events by an external committee. A total of 7 patients died during the study, 3 in the semaglutide group and 4 in the placebo group, for an incidence of 1.1% and 1.5%, respectively (Table 3). None of the deaths in the semaglutide group were attributed to CV causes [50]. The incidence of HF events was low. It occurred in 0.4% (*n* = 1) of the participants in the semaglutide group and in 4.5% (*n* = 12) of the participants in the placebo group (Table 3) [50].

**Table 3:** Key information of the STEP-HFpEF trial [50]

STEP-HFpEF		
**Class &** **Cardiovascular (CV) outcomes**	**Estimated difference or ratio (95% CI)**	***p-value***
**Dual primary endpoints**		
Change in KCCQ-CSS from baseline to week 52 (points)	7.8 (4.8 to 10.9)	< 0.001
Change in body weight from baseline to week 52 (%)	-10.7 (-11.9 to -9.4)	< 0.001
**Confirmatory secondary endpoints**		
Change from baseline to week 52 in 6-minute walk distance (m)	20.3 (8.6 to 32.1)	< 0.001
Change from baseline to week 52 in CRP level (%)	0.61 (0.51 to 0.72)	< 0.001
Hierarchical composite endpoint (crude % of wins)	1.72 (1.37 to 2.15)	< 0.001
**Adverse events**	**Event rate (%) active vs. placebo group**	***p-value***
**Serious adverse events**	13.3 vs. 26.7	< 0.001
Cardiac disorder	2.7 vs. 11.3	< 0.001
Gastrointestinal disorders	2.7 vs. 2.7	1.0
**Adjudicated events**		
Death from any cause	1.1 vs. 1.5	n.a.
CV death	0 vs. 0.4	n.a.
HF event	0.4 vs. 4.5	n.a.

CI, confidence interval; CV, cardiovascular; HF, heart failure; HR, hazard ratio; KCCQ-CSS, Kansas City Cardiomyopathy Questionnaire clinical summary score; n.a., not applicable

## Novel cholesterol-lowering medication

### CLEAR outcomes

The CLEAR Outcomes trial [55] evaluated the effects of bempedoic acid (180 mg/daily) on adverse CV events in patients at high risk for CVD, who were unable or unwilling to take statins owing to unacceptable adverse effects [55].

A total of 13,970 individuals aged 18 to 85 years were randomly assigned to bempedoic acid (*n* = 6992) or placebo (*n* = 6978). Participants were eligible if they had a clinical feature that placed them at high risk for a cardiovascular event (30%; *n* = 4206) or a previous cardiovascular event (70%; *n* = 9764). Patients who were receiving a very low average daily statin dose without unacceptable adverse effects or other lipid-lowering therapies could be enrolled [55]. The mean LDL cholesterol (LDL-C) level at baseline was 139 mg/dL (3.59 mmol/L). Among the patients, 45.6% (*n* = 6373) had diabetes and 22.7% were taking very low dose statin therapy [55].

The primary endpoint, assessed in a time-to first-event analysis, was a four-point composite of major adverse cardiovascular events (4P-MACE), defined as death from cardiovascular causes, nonfatal MI, nonfatal stroke, or coronary revascularization. The key secondary endpoints, assessed in a time-to first-event analysis and tested in hierarchical order, included a 3P-MACE of death from CV causes, nonfatal stroke, or nonfatal MI; fatal or nonfatal MI; coronary revascularization; fatal or nonfatal stroke; death from CV causes; and death from any cause. Additional secondary endpoints established for this trial have been further described [55].

At baseline, the mean LDL-C level was 139.0 mg/dL (3.59 mmol/L) in both the treatment and placebo groups [55]. After 6 months of treatment with bempedoic acid, the average level of LDL-C in this patient group decreased by 21.1% from baseline to 107.0 mg/dL (2.77 mmol/L), whereas in the placebo group, a mean reduction of 0.8% to 136.0 mg/dL (3.52 mmol/L) was observed. During the same period, a 21.6% reduction in the CRP level was observed in the bempedoic acid group compared to the placebo group [42].

After 12 months of treatment with bempedoic acid, the mean change in the HbA1c level of patients with inadequately controlled T2D [HbA1c > 7% (53 mmol/mol) at baseline] was − 0.04% in the bempedoic acid group compared to -0.01% in the placebo group [55].

Following a median 40.6 months trial follow-up, a primary endpoint MACE event was observed in 11.7% (*n* = 819) of the patients in the bempedoic acid group and in 13.3% (*n* = 927) of the patients of the placebo group, resulting in a significant 13% lower MACE incidence in the treatment group (hazard ratio (HR) 0.87; 95% confidence interval (CI) 0.79 to 0.96; *p* = 0.004) (Table 4) [55]. The risk of events of the first key secondary endpoint, the composite of 3P-MACE, was also 15% lower in the bempedoic acid group than in the placebo group (8.2% [*n* = 575] vs. 9.5% [*n* = 663]; HR 0.85; 95% CI 0.76 to 0.96; *p* = 0.006)(Table 4) [55]. The other hierarchical tested two secondary endpoints also showed significant benefits with bempedoic acid over placebo: fatal and nonfatal MI [3.7% (*n* = 261) vs. 4.8% (*n* = 334); HR 0.77; 95% CI 0.66 to 0.91; *p* = 0.002] and coronary revascularization [6.2% (*n* = 435) vs. 7.6% (*n* = 529); HR 0.81; 95% CI 0.72 to 0.92; *p* = 0.001] (Table 4) [55]. The incidences of the other key secondary endpoints: fatal or nonfatal stroke, death from cardiovascular causes, and death from any cause, were not significantly different between the two groups (Table 4), and similar results were observed for additional defined secondary endpoints [55].

The overall incidence of adverse events, musculoskeletal adverse events and other adverse events of interest including new onset of diabetes, worsening hyperglycemia, hypoglycemia, and metabolic acidosis did not differ meaningfully between both groups (Table 4). However, the bempedoic acid group had higher incidences of elevated hepatic-enzyme levels, renal impairment, hyperuricemia, gout and cholelithiasis (Table 4) [55].

**Table 4:** Key information of the CLEAR Outcomes trial [55]

CLEAR Outcomes		
**Class &** **Cardiovascular (CV) outcomes**	**HR (95% CI)**	***p-value***‡
**Primary efficacy endpoint**		
Composite of 4P-MACE: CV death, nonfatal MI, nonfatal stroke, or coronary revascularization	0.87 (0.79 to 0.96)	0.004
**Key secondary efficacy endpoints**		
Composite of 3P-MACE: CV death, nonfatal stroke, or nonfatal MI	0.85 (0.76 to 0.96)	0.006
Fatal or nonfatal MI	0.77 (0.66 to 0.91)	0.002
Coronary revascularization	0.81 (0.72 to 0.92)	0.001
Fatal or nonfatal stroke	0.85 (0.67 to 1.07)	0.16
CV death	1.04 (0.88 to 1.24)	
Death from any cause	1.03 (0.90 to 1.18)	
**Adverse events of special interest**	**Event rate (%) active vs. placebo group**	
Any adverse event that started or worsened after the first dose of a trial agent	86.3 vs. 85.0	
Any muscle disorder adverse event	15.0 vs. 15.4	
Myalgia	5.6 vs. 6.8	
New onset diabetes in patients without diabetes at baseline	16.1 vs. 17.1	
Worsening hyperglycemia	22.7 vs. 23.1	
Hypoglycemia	4.3 vs. 3.8	
Metabolic acidosis	0.2 vs. 0.2	
Elevated hepatic-enzyme level	4.5 vs. 3.0	
Renal impairment	11.5 vs. 8.6	
Hyperuricemia	10.9 vs. 5.6	
Gout	3.1 vs. 2.1	
Cholelithiasis	2.2 vs. 1.2	

‡ p-values after the first nonsignificant p-value are not presented

CI, confidence interval; CV, cardiovascular; HR, hazard ratio; MACE, major adverse cardiovascular event; MI, myocardial infarction

## Key topics discussed during the 9th CVOT Summit

### Guidelines

#### Evidence production and guideline’s development

Typically, recommendations in clinical guidelines have been formulated based on evidence from CVOTs and outcomes of systematic reviews, including meta-analyses of all readily available randomized controlled trials (RCTs). However, the field of diabetology is advancing so rapidly that the magnitude of the data provided by RCTs makes it impractical to use this approach for risk-benefit determinations when there are multiple treatment options for patients with the same disease [65]. Hence, another statistical technique that combines both direct and indirect evidence in a single analysis is currently being employed for comparing multiple interventions for a specific condition: network meta-analysis (NMA). In 2023, an NMA and systematic review on the benefits and harms of medical treatments for adults with T2D, validated through CVOTs, was published [66]. The analysis identified 816 trials with a total of 471 038 patients, evaluated 13 different drug classes and confirmed the benefits of SGLT2is and GLP-1 RAs in reducing CV death, nonfatal MI, HHF and end-stage kidney disease [66]. According to the same analysis, finerenone is likely to reduce HHF, end-stage renal disease and possibly CV death [66]. Only GLP-1 RAs were found to reduce nonfatal stroke and SGLT2is were found to be superior to other drugs in reducing end-stage kidney disease [66]. Reported adverse events were largely drug class-specific (e.g., genital infections with SGLT2is; serious gastrointestinal adverse events with tirzepatide and GLP-1 RAs; hyperkalemia requiring hospitalization with finerenone). The study showed that tirzepatide is likely to result in the greatest weight loss, while basal insulin and thiazolidinediones are likely to result in the greatest weight gain [66]. In addition, the absolute benefit of SGLT2is, GLP-1 RAs, and finerenone in people with T2D was found to vary according to baseline risk for CV and kidney outcomes [66].

Production of reliable evidence in a research field that requires constant information update can be costly and time consuming [67]. The Alliance for Living Evidence (www.aliveevidence.org) is a global initiative of the Future Evidence Foundation. It is collaborating with a range of partners to continuously update reviews of science and deliver timelier, trustworthy, and affordable evidence for better decision-making.

#### Update on HF and CV risk management in guidelines

In 2023 a focused update of the 2021 European Society of Cardiology (ESC) guidelines for the diagnosis and treatment of acute and chronic HF [68] as well as new ESC guidelines for the management of CVD in patients with diabetes [69] were published. Both guidelines reflect in their recommendations new evidence from recent CVOTs with SGLT2is [30, 31] and finerenone [35–37].

The focused update of the ESC HF guidelines [68] recommends a SGLT2i (dapagliflozin or empagliflozin) in patients with HF with mildly reduced ejection fraction (HFmrEF) or with HFpEF, as well as in patients with T2D and CKD, to reduce the risk of HHF or CV death. Finerenone is recommended in patients with T2D and CKD to reduce the risk of HHF [68]. Intravenous iron supplementation is recommended to alleviate HF symptoms and improve quality of life in symptomatic patients with heart failure with reduction fraction (HFrEF) or HFmrEF and iron deficiency [68].

The 2023 ESC guidelines on the management of CVD in patients with diabetes [69] recommend systematic screening for diabetes in all individuals with CVD, using fasting glucose and/or HbA1c and introduce a novel 10-year CVD risk score (SCORE2-Diabetes) specific for patients with T2D without atherosclerotic cardiovascular disease (ASCVD) or severe target-organ damage (TOD). On the other hand, patients with T2D and ASCVD are recommended to initiate treatment with GLP-1 RAs and SGLT2is to reduce CV risk, independent of glucose control and in addition to antiplatelet, anti-hypertensive or lipid-lowering therapy [69]. In patients with T2D and CKD it is recommended to add a SGLT2i (canagliflozin, dapagliflozin or empagliflozin) and finerenone to standard of care, to reduce CV and kidney failure risk [69]. The guidelines also address the treatment of patients with HF and T2D with glucose-lowering medications. An SGLT2i is recommended to reduce HF-related outcomes in all patients with T2D and HF (HFpEF, HFmrEF, HFrEF), independent of HbA1c level or concomitant use of glucose-lowering medications. Moreover, the addition of other glucose-lowering agents with neutral effects on HF, such as a GLP-1 RA, sitagliptin or linagliptin, metformin, or insulin glargine or degludec, should be considered if additional glycemic control is needed. Pioglitazone and saxagliptin are not recommended due to their association with an increased risk for HHF as shown in CVOTs [69].

#### Towards the recognition of treatment disparities and the inclusion of patient-reported outcomes (PROs) in clinical guidelines

Although significant advances have been made in diabetes management, population-specific efficacy and safety information has yet to be incorporated into clinical guidelines. In general, racial, and ethnic groups are underrepresented in clinical trials, and recommendations for diabetes practice are based on the assumption of overall broad-based drug efficacy. But this may not always be the case. A recent meta-analysis of CVOTs with SGLT2is and GLP-1 RAs [70] found substantial racial/ethnic disparities in the cardiorenal effects of SGLT2is and GLP-1 RAs in patients with T2D, with consistent benefits observed among white and Asian populations and consistent lack of benefits in black populations [70]. However, the observed trends in the black populations may be due to lower enrollment in clinical trials (2.4-8,3%) and wide variability in outcomes [70]. Also not to be overlooked is the impact of the social determinants of health on disparities in the equity of care, such as access to diabetes technology.

Another important aspect to consider in guidelines is the inclusion of patient-reported outcomes (PROs). In fact, diabetes is unique among chronic diseases in the extent to which clinical outcomes are controlled by the patient. Achieving and maintaining control of the condition is challenging and requires the individual to deal with a wide range of behaviors and cognitions, resulting in a high burden of psychological distress [71]. This means that the patients’ experience and perspective on the impact of attempting to control these outcomes can significantly influence their health-related quality of life. Therefore, when evaluating the effectiveness of a diabetes treatment, it is important to consider patient needs and feedback as well as clinical endpoints, a position supported by the IDF [72].

### Importance of screening, identification, and management of CKD in primary care

CKD is a progressive condition that affects > 10% of the general population worldwide [73]. Globally, diabetes is the leading cause of CKD morbidity [74]. Diabetic nephropathy is asymptomatic in early stages, making it difficult to control. Early identification, risk stratification, and treatment can reduce both morbidity and mortality rates from CKD and its related complications, like CVD. The American Diabetes Association (ADA) and Kidney Disease: Improving Global Outcomes (KDIGO) recommend annual screening of patients with diabetes for CKD, beginning 5 years after diagnosis of T1D and diagnosis of T2D [75]. Screening should be made by measuring both the urine albumin-to-creatinine ratio (UACR) and the estimated glomerular filtration rate (eGFR) [75].

Primary care can play an important role in the early detection and management of CKD, as well as in patient education. Nevertheless, a survey of primary care professionals in Europe [76] found that only 32.4% were fully confident in interpreting UACR results. Currently, UACR can also be determined by point-of-care testing (POCT) [77]. The survey also revealed that there is an unmet need for more information on screening, detection, and management of CKD [76]. Similar results are being observed in China and were reported at the CVOT Summit. The International Society of Nephrology (ISN) has made available online a new clinical one-pager on “Chronic Kidney Disease (CKD) Management-Early Identification and Intervention in Primary Care”- which has been endorsed by primary care organizations like PCDE and World family doctors Caring for people (Wonca) Europe [78].

### Advances in the management of CKD with SGLT2is and nsMRAs

KDIGO published in 2022 clinical practical guidelines for diabetes management in CKD [79], recommending SGLT2is as first-line drug therapy and considering finerenone for additional risk-based therapy in individuals with persistent albuminuria despite use of ACEis or ARBs. GLP-1 RAs are not currently indicated to improve kidney outcomes, although there is accumulating evidence from CVOTs of a kidney and cardioprotective effect in people with T2D and CKD [80]. However, since GLP-1 RAs effectively reduce HbA1c and CKD is associated with CVD, the guideline recognizes the potential of GLP-1 RAs as adjuncts to metformin and SGLT2i [79].

The benefit of SGLT2is to delay CKD progression are consistent in patients with and without T2D [81]. Promising results in reducing UACR in CKD patients with an eGFR > 20 ml/min/1.73 m² and an UACR of 150–5000 mg/g with a combination treatment of dapagliflozin and zibotentan, an endothelial A receptor antagonist, have been reported [82]. EMPA-KIDNEY results suggest that empagliflozin may slow CKD progression in patients with CKD and T1D [34].

There have also been advances in the management of CKD and comorbidities with nsMRAs. Ocedurenone has been shown to reduce systolic blood pressure in patients with CKD stage 3b/4 with uncontrolled or resistant hypertension [83]. A FIDELITY subgroup analysis [84] showed that finerenone consistently reduced albuminuria and eGFR decline in patients with CKD stage 4 and T2D, but the effects on the composite kidney outcome were not consistent over time. The FINE-ONE trial [82] is ongoing and is evaluating the effects of finerenone in patients with CKD and T1D.

### Diabetes, obesity, liver disease and cancer

Diabetes and obesity are well-established risk factors for several human cancers. The combined effect of diabetes and high BMI (25 kg/m²) as independent risk factors was responsible for 5.7% of all incident cancers in 2012, accounting for 24.5% of all liver cancer cases and 38.4% of all endometrial cancer cases [85]. In 2019, high BMI was the third leading risk factor for risk-attributable cancers and DALYs for both sexes, after smoking and alcohol consumption [86].

T2D and obesity share common metabolic pathways which may contribute to the development of cancer. For instance, the adipose tissue is an organ that produces adipokines, such as leptin, which is involved in the regulation of energy balance and has receptors widely expressed in the peripheral tissue, including pancreatic beta-cells. This hormone is commonly elevated in individuals with obesity and diabetes due to leptin resistance without any reduction in appetite [87]. Association of leptin with metastasis and cancer aggressiveness has been shown and attributed to its mitogenic and proinflammatory effects [88].

Moreover, obesity-associated insulin resistance and hyperinsulinemia are also associated with inflammation and carcinogenesis, through the activation of insulin and IGF-1 receptor signal transduction pathways, as demonstrated for pancreatic cancer and hepatocellular carcinoma (HCC) [89, 90].

HCC is the most prevalent form of liver cancer and may develop from progressive fibrosis in fatty liver disease. Hepatic fibrosis is triggered by inflammation and is a key stage in the progression of organ failure and death [91].

To date, no pharmacologic agent is approved for the treatment of NASH. However, Phase III clinical trials with resmetirom, a thyroid hormone receptor beta agonist, showed positive results [92] and resmetirom has been submitted to the FDA for approval, with a decision expected in March 2024.

Further trials with efruxifermin [93] and pegozafermin [94], fibroblast growth factor 21 (FGF21) agonists and with the glucose-lowering agents semaglutide (GLP-1 RA) [95] and tirzepatide (GIP/GLP-1 RA) [96] are ongoing.

Indeed, both NAFLD and NASH have been acknowledged as metabolic dysfunctions and have been renamed metabolic dysfunction associated steatotic liver disease (MASLD) and metabolic dysfunction associated steatohepatitis (MASH), respectively [97]. The nomenclature has been changed in 2023, to avoid potential stigmatizing language and to abridge the presence of at least 1 of 5 cardiometabolic risk factors in MASLD [98].

### Harnessing incretin and glucagon physiology for diabetes and obesity management

The quest for better diabetes and weight loss medicines hasn’t stopped with GIP/GLP-1 RAs. Research on the physiology of GIP and the GIP receptor (GIPR) has shown that GIP decreases body weight and food intake without affecting meal frequency via GIPR signaling in the central nervous system [99]. More recently, GIP and GIPR/GLP-1 R co-agonists were shown to decrease body weight and food intake via signaling through GABAergic GIPR neurons [100]. On the other hand, glucagon, which is secreted from pancreatic alpha-cells in response to blood glucose levels, is also known to reduce food intake, increase energy expenditure and promote hepatic fatty acid oxidation [101]. Hence, the current focus of research on diabetes and obesity is on the development of triple agonists that target all three receptors, specifically GIP/GLP-1/glucagon receptor agonists.

In the year 2023, the results of two Phase II clinical trials with the triple agonist retatrutide in T2D [102] and obesity [103] were published. These findings have demonstrated that subcutaneous retatrutide administered at a dose of 12 mg can result in a mean change in HbA1c of 2.2% compared to baseline and a reduction in body weight of 24.2% [102, 103].

Also in 2023, the results of the PIONEER PLUS trial were published [104]. PIONEER PLUS evaluated the effects of high-dose oral semaglutide (50 mg) on both glycemic and weight control. It showed that a 2.2% reduction in HbA1c and a 9.2% reduction in body weight could be achieved, relative to a mean baseline HbA1c of 9% (75 mmol/mol) and a mean baseline body weight of 96.4 kg [104]. Moreover, promising results with orforglipron [105], a nonpeptide oral GLP-1 RA were also presented at the CVOT Summit. At week 26, there was a significant change in mean placebo adjusted HbA1c (-1.67%) and bodyweight (-7.9 kg) [105].

### Patient-physician communication in the management of diabetes and obesity

Patients with diabetes report experiencing stigma in many life domains [106], a concern shared by people with obesity [107]. The psychological impact of living with a stigmatized condition is significant and may be a barrier to optimal self-care. Patient-physician communication plays an important role in diabetes and obesity management, and careful and constructive language should be used in conversations with people living with obesity, as well as a positive approach to recommending weight management programs [107, 108].

### New developments in the treatment of T1D

The IDF reported that in 2022, approximately 8.75 million people were living with T1D [109], and its prevalence is estimated to increase up to 17.4 million by 2040 [110]. T1D is an autoimmune disease that can be triggered by environmental factors, such as viral infection, diet, and growth (in children), in individuals who are genetically susceptible [111]. Screening in childhood and detecting the disease earlier in its course leads to a more favorable clinical outcome at symptom onset [112]. In this regard, the Global Platform for the Prevention of Autoimmune Diabetes (GPPAD) (https://www.gppad.org/) is conducting two primary prevention studies: the “Primary prevention with oral insulin” (POInT) and the ”Supplementation with *Bifidobacterium infantis* for Mitigation of Type 1 Diabetes Autoimmunity” (SINT1A) in children with an increased risk of developing T1D.

In secondary prevention, a breakthrough was achieved with the FDA approval in November 2022 of the first disease-modifying therapy, teplizumab, to delay the progression of autoimmunity in patients aged ≥ 8 years with stage 2 T1D (preclinical disease). Teplizumab, a humanized monoclonal antibody, targets T-lymphocytes carrying CD3 on their surface, delivering a partial agonistic signal and inhibiting the immune attack on pancreatic beta-cells. In this early phase of T1D, a single 14-day course of teplizumab has been demonstrated to defer the progression from stage 2 to stage 3 by 59.6 months and enhance beta-cell function [113]. In PROTECT [114], a subsequent 78-week Phase III RCT in children and adolescents (8–17 years old) newly diagnosed with stage 3 T1D, teplizumab has been shown to halt beta-cell destruction as measured by quantifying the level of stimulated C-peptide in the blood following a 4-hour mixed meal tolerance test. In this study, only patients with a peak C-peptide ≥ 0.2 pmol/mL at baseline were considered eligible [114]. Participants received teplizumab (*n* = 217) or placebo (*n* = 111) in two 12-day courses separated by 26 weeks, with the primary endpoint being the change from baseline in stimulated C-peptide levels at week 78 [114]. Stimulated C-peptide levels were then observed to be significantly higher in the teplizumab group than in the placebo group (least squares mean difference 0,13 pmol/mL; 95% CI 0.09 to 0.17; *p* < 0.001) [114], mainly due to the effect of the first treatment course. Furthermore, 94.9% of the patients treated with teplizumab maintained a peak C-peptide level ≥ 0.2 pmol/mL, compared to 79.2% in the placebo group [114], indicating a protective effect of teplizumab on pancreatic beta-cells. Noteworthy, teplizumab-treated patients required lower doses of insulin than placebo-treated patients [114] to achieve the same glycemic goal [HbA1c < 7% (53 mmol/mol)], also reflecting the protective effect on functional active beta-cells at stage 3 T1D (symptom onset). The groups did not differ significantly in the key secondary endpoints (HbA1c, TIR, clinically important hypoglycemic events) [114]. The most common adverse events leading to study discontinuation were protocol defined liver function test elevations (teplizumab 6.9%; placebo 2.7%) and cytokine release syndrome (teplizumab 1.8%; placebo 0%) [114].

### Microvascular complications

Diabetic peripheral neuropathy (DPN) is a major microvascular complication in people with diabetes. The prevalence of DPN in T1D was determined to be 23.5% during a follow-up period of 7.3 years, with women exhibiting the highest frequency of developing painful DPN [115]. Among guideline-recommended first-line analgesic monotherapies, the OPTION-DM trial [116] found that amitriptyline, duloxetine and pregabalin had similar efficacy and that combination treatment was well tolerated and led to improved pain relief in patients with suboptimal pain control on monotherapy. Pregabalin was the best tolerated monotherapy associated with the least discontinuation due to treatment emergent adverse events [116].

Recently, a consensus recommendation on screening, diagnosis, and management of diabetic sensorimotor polyneuropathy (DSPN) in clinical practice was published [117]. As pathogenetically oriented pharmacotherapies the antioxidant alpha-lipoic acid (ALA) and benfotiamine, a thiamine derivative (prodrug) and AGE inhibitor, are approved for clinical use in several countries. Both compounds have favorable safety profiles, even with long-term treatment [117]. Treatment with 600 mg oral administered ALA once daily for 5 weeks and twice daily for 6 months reduced pain, paresthesia, and numbness in patients with DSPN [118, 119]. In addition, in patients with mild to moderate DSPN, neuropathic deficits were improved after 4 years of treatment with 600 mg ALA once daily [120]. Twice-daily treatment with 300 mg benfotiamine for 6 weeks improved neuropathic symptoms as assessed by Neuropathy Symptom Score (NSS) in patients with symmetrical, distal diabetic neuropathy [121].

Nonetheless, enhanced glucose control, lifestyle modification, control of CV risk factors, and utmost attention to foot care, remain fundamental measures for the prevention and management of DPN [117].

### CGM metrics and cognitive disorders

Aside from CVD, T2D is also associated with dementia [122]. It has been observed that the HR increases with higher glucose levels [122]. People diagnosed with diabetes before the age of 60 were found to have a threefold increased risk of dementia [123] and in adults with T2D aged 70 years or older using CGM, a higher time above range (TAR) was found associated with cognitive impairment of both executive and working memories [124]. Importantly, glycemic variability and time below range (TBR) were not found associated with any cognitive function [124]. Furthermore, subcortical brain damage has been found associated with hyperglycemia [125, 126] independently of lifestyle and CV risk factors [126], but not with HbA1c [126]. Thus, evidence is accumulating to support the use of CGM in people with T2D. Not only because time in range (TIR) is a surrogate marker for all-cause and CV mortality [127], but also because analysis of CGM metrics may be helpful for early intervention and prevention of dementia in the future.

## Conclusions

The 9th CVOT Summit: Congress on Cardiovascular, Kidney, and Metabolic Outcomes provided an interactive and multidisciplinary platform to discuss key results from recently published trials with SGLT2i (DAPA-MI), GLP-1 RA (SELECT and STEP-HFpEF), and bempedoic acid (CLEAR Outcomes). Important advances have been made in the treatment of CVD, HF and CKD in diabetes or obesity, and a breakthrough was achieved in the secondary prevention of T1D in 2023. Further developments in the treatment of MASH and obesity are expected in 2024. At the CVOT Summit these and other topics including guideline development, enrollment of ethnic/racial groups in clinical trials, and the importance of patient-physician communication and PROs were discussed by a broad audience of both specialists and primary care physicians. The 10th CVOT Summit will be held virtually on December 5–6, 2024 (http://www.cvot.org).

## Data Availability

No datasets were generated or analysed during the current study.

## Abbreviations

ACEis: Angiotensin-converting-enzyme inhibitors
ADA: American Diabetes Association
AGE: Advanced glycation endproduct
AGP: Ambulatory Glucose Profile
ALA: Alpha-lipoic acid
ARBs: Angiotensin-receptor blockers
ASCVD: Atherosclerotic cardiovascular disease
ATP: Adenosine triphosphate
BMI: Body mass index
CGM: Continuous glucose monitoring
CI: Confidence interval
CKD: Chronic kidney disease
CRP: C-reactive protein
CV: Cardiovascular
CVD: Cardiovascular disease
CVOT: Cardiovascular outcome trial
DALYs: Disability-adjusted life years
DKD: Kidney disease due to diabetes
DPN: Diabetic peripheral neuropathy
DPP-4i: Di-peptidyl peptidase 4 inhibitor
DSPN: Diabetic sensorimotor polyneuropathy
eGFR: Estimated glomerular filtration rate
ESC: European Society of Cardiology
FDA: U.S. Food and Drug Administration
GABA: Gamma-aminobutyric acid
GIP: Glucose-dependent insulinotropic polypeptide
GLP-1 RA: Glucagon-like peptide 1 receptor agonist
HbA1c: Glycated hemoglobin A1c
HCC: Hepatocellular carcinoma
HF: Heart failure
HFmrEF: Heart failure with mildly reduced ejection fraction
HFpEF: Heart failure with preserved ejection fraction
HFrEF: Heart failure with reserved ejection fraction
HHF: Hospitalization for heart failure
HMC-CoA: 3-hydroxy-3-methylglutaryl coenzyme A
HR: Hazard ratio
IDF: International Diabetes Federation
KCCQ-CSS: Kansas City Cardiomyopathy Questionnaire clinical summary score
LDL: Low-density lipoprotein
LDL-C: LDL cholesterol
LV: Left ventricular
LVEF: Left ventricular ejection fraction
MACE: Major adverse cardiovascular event
MASLD: Metabolic dysfunction associated steatotic liver disease
MASH: Metabolic dysfunction associated steatohepatitis
MI: Myocardial infarction
NAFLD: Nonalcoholic fatty liver disease
NASH: Nonalcoholic steatohepatitis
nsMRA: Nonsteroidal mineralocorticoid receptor antagonist
NSS: Neuropathy Symptom Score
NYHA: New York Heart Association
PCDE: Primary Care Diabetes Europe
PROs: Patient-reported outcomes
RCT: Randomized controlled trial
SGLT2i: Sodium-glucose cotransporter-2 inhibitor
T1D: Type 1 diabetes
T2D: Type 2 diabetes
TAR: Time above range
TBR: Time bellow range
TIR: Time in range
TOD: Target-organ damage
UACR: Urine albumin-to-creatinine ratio
